# Periodontal Medicine 2.0: a framework for evidence-calibrated, integrated and patient-centred care

**DOI:** 10.3389/fdmed.2026.1907427

**Published:** 2026-09-29

**Authors:** Marde Shraddha Kashinath, Geeta Mandavilli, Prathyusha Chava

**Affiliations:** 1 Department of Periodontics, ESIC Dental College and Hospital, Kalaburagi, India; 2 Aspen Dental Management Inc., Syracuse, NY, United States

**Keywords:** artificial intelligence, implementation science, integrated healthcare, oral-systemic health, patient-centred care, periodontal medicine, precision periodontology

## Abstract

Periodontal medicine has generated substantial evidence that periodontal and systemic health are biologically, behaviourally, and socially interconnected. Yet a persistent translational gap remains: knowledge of oral-systemic relationships has advanced faster than the systems required to convert this knowledge into coordinated, patient-centred care. Dentistry and medicine continue to operate within fragmented professional structures, limiting opportunities for prevention, early risk identification, and longitudinal disease management. We propose Periodontal Medicine 2.0 (PM2.0), an author derived conceptual hypothesis that reimagines periodontal medicine beyond the study of oral-systemic associations toward evidence-calibrated translation and integrated care. Its proposed contribution lies in the clinically significant interactions amongst the four principal domains centred on the periodontal patient: integrated oral-systemic screening, interprofessional collaborative care, precision periodontal medicine, and population health and policy integration. Digital health and AI, patient engagement, health literacy, equity, ethics and implementation science are positioned as cross-cutting enablers. PM2.0 proposes that these domains should interact according to evidence maturity, clinical relevance and implementation readiness, rather than through uniform integration. The framework distinguishes association from causality, screening from diagnosis, diagnostic performance from clinical utility, and technological capability from demonstrated patient benefit. It proposes a risk based, longitudinal pathway linking periodontal assessment with selective risk identification, coordinated care, individualized management and evaluation. The central hypothesis is that appropriately connected and evidence-calibrated functions may generate greater translational value than fragmented approaches. This proposition is testable through clinical, patient reported, implementation, economic, safety and equity outcomes. PM2.0 is therefore presented not as an established historical stage, clinical guideline or validated model of care, but as a literature-informed hypothesis for the future organization of periodontal medicine- from association to evidence based action, and from action to demonstrable impact.

## 1 Introduction

The conceptual boundaries of periodontology have progressively expanded from the local management of periodontal inflammation toward recognition of its relationship with broader biological, behavioural and social determinants of health. Early periodontal medicine was shaped by evidence suggesting that periodontal inflammation, microbial dissemination, and host mediated responses could have biological consequences beyond the oral cavity (1, 2). Subsequent epidemiological, mechanistic, and clinical research identified relationships between periodontitis and systemic conditions including diabetes, cardiovascular disease, pregnancy related outcomes, and other chronic disorders, establishing oral-systemic health as an important area of interdisciplinary investigation (3–6).

This broader perspective does not diminish the central role of periodontal treatment. Effective control of periodontal inflammation remains the foundation of periodontal care, with treatment guided by disease severity, risk factors, treatment response and patient needs (2). Periodontal Medicine 2.0 (PM2.0) builds on this established therapeutic foundation by examining how validated oral-systemic knowledge can be translated into coordinated and patient-centred care.

The scientific basis of periodontal medicine has subsequently matured through advances in periodontal biology, host response research, clinical trials, disease classification, and international consensus initiatives (4–8). The relationship between periodontitis and diabetes provides an important example of clinically meaningful oral-systemic interaction, supported by substantial biological, epidemiological, and interventional evidence (9–11). In contrast, evidence concerning cardiovascular disease, pregnancy outcomes, neurodegenerative disorders, and other systemic conditions varies in strength and remains heterogeneous (12–25). Recognizing these differences is essential if periodontal medicine is to progress beyond association without overstating the certainty of existing evidence (26).

The central challenge, therefore, is no longer simply to establish that oral and systemic health are related. The more consequential question is which relationships are sufficiently established to inform action, for which patients, through which care pathways, and with what measurable benefit. This shift from knowledge generation toward evidence-calibrated translation represents the conceptual space in which the present framework is positioned.

Translation, however, occurs within healthcare systems that frequently remain structurally fragmented. Dental and medical services may operate through separate professional structures, clinical records, referral pathways and educational systems. Such fragmentation can constrain communication, limit opportunities for coordinated prevention, and make clinically relevant oral-systemic information difficult to translate into appropriate healthcare action (27–31). At the policy level, international initiatives have increasingly emphasized the integration of oral health into universal health coverage and broader health-system strategies, providing an important foundation for more connected models of care (32–36).

Meanwhile, precision medicine, digital health, artificial intelligence, microbiome science, biomarkers, and patient-centred approaches are creating new possibilities for periodontal healthcare. These developments could support more individualized risk assessment, improved information exchange, longitudinal monitoring, patient engagement, and population level prevention. However, biological plausibility, technical capability, or diagnostic performance should not be equated automatically with clinical utility or improved patient outcomes. These distinctions are particularly important when considering the future organization of periodontal care.

Against this background, we propose “PM2.0” as an author derived conceptual framework for addressing the translational gap between periodontal knowledge and integrated healthcare.

The proposed contribution is architectural rather than component based: PM2.0 defines how these established functions interact within a periodontal specific evidence to impact pathway, from risk identification and coordinated action to individualized management, longitudinal monitoring, and population level implementation. Its novelty therefore lies in the interdependent domains, evidence calibration, and testable integration of these domains.

## 2 Conceptual methodology and scope

### 2.1 Study design

This article used a structured conceptual synthesis and framework development methodology rather than a systematic review methodology to develop PM2.0. The objective was not to estimate treatment effects or establish pooled associations, but to identify relevant established and emerging concepts and organize them into a coherent, periodontal specific and hypothesis generating framework. The methodology comprised the following linked stages.

### 2.2 Literature identification

Literature was identified through PubMed/MEDLINE, Scopus, Web of Science, and Google Scholar from January 1, 1996, through July 31, 2026, supplemented by publications and policy documents from the World Health Organization (WHO), European Federation of Periodontology (EFP), American Academy of Periodontology (AAP), and relevant international periodontal, medical, and public health organizations.

Searches were performed using combinations of terms related to “periodontitis,” “periodontal medicine,” “oral-systemic health,” “integrated healthcare,” “precision dentistry,” “digital health,” “artificial intelligence,” “implementation science,” and “population oral health.” Publications were selected based on relevance to periodontal-systemic relationships, healthcare integration, emerging technologies, and health system implementation. Foundational literature and contemporary evidence were considered, with greater emphasis on systematic reviews, consensus reports, clinical guidelines, major reviews, and authoritative policy documents. Contemporary literature was specifically considered for rapidly developing areas including precision medicine, biomarkers, microbiome research, digital health, artificial intelligence (AI), implementation science, healthy ageing, and the oral-brain axis.

Reference lists of key reviews, guidelines, consensus documents, and conceptual papers were also examined to identify relevant frameworks and foundational sources.

### 2.3 Evidence selection and calibration

Evidence was selected according to relevance, authority, conceptual contribution and translational importance. No formal quality appraisal or risk of bias assessment was undertaken because the objective was conceptual framework development rather than estimation of intervention effects. Evidence was therefore used to inform the identification of domains and their conceptual calibration rather than to generate pooled estimates or comparative effectiveness conclusions.

For framework development, evidence was considered according to its degree of maturity and readiness for translation, with distinctions made between established, emerging, research stage and conceptual/future evidence.

### 2.4 Identification of existing frameworks

Existing models were identified through targeted searches concerning periodontal medicine, oral-systemic health, integrated healthcare, interprofessional collaboration, precision oral health, person-centred care, digital health, implementation science, population health and health system integration.

Frameworks were compared according to six conceptual dimensions:

- Their principal purpose
- Healthcare level addressed
- Domains included
- Relationships between domains
- Periodontal specificity
- Ability to connect evidence with implementation and measurable outcomes.

The comparison examined whether existing approaches provide a periodontal specific architecture linking these functions through explicit relationships. Existing models generally emphasize one or more components of the proposed framework. These approaches provide important conceptual and operational foundations, but they differ in scope, purpose, healthcare level and degree of periodontal specificity.

The unresolved conceptual gap identified through this comparison was therefore not the absence of individual components, but the absence of an explicitly periodontal centred architecture that specifies how these functions interact across the pathway from risk identification to coordinated action, individualized periodontal management, longitudinal monitoring and system level implementation. In particular, existing approaches do not necessarily define evidence maturity as a gate for progression between these functions, nor do they consistently connect clinical utility, implementation feasibility, patient benefit, equity and sustainability within a single periodontal specific pathway.

### 2.5 Derivation and qualification of the principal domains

Candidate domains were identified from recurring themes in the literature and grouped according to their principal function in translating periodontal knowledge into healthcare impact.

A domain was retained as a principal component when it demonstrated: (1) an established or developing evidence base; (2) direct relevance to periodontal or oral-systemic health; (3) a distinguishable translational function; and (4) meaningful relationships with other domains.

This process generated four principal functional domains:

1. Integrated oral-systemic screening
2. Interprofessional collaborative care
3. Precision periodontal medicine
4. Population health and policy integration.

Digital health and AI, patient engagement, health literacy, equity, ethics and implementation science were treated as cross-cutting enablers because they influence multiple domains.

### 2.6 Conceptual mapping and coherence

The domains were iteratively mapped according to three questions:

What does the domain accomplish? What does it require from other domains? What level of impact can it influence?

This generated evidence to impact architecture in which:

Screening identifies actionable risk → collaborative care converts risk into coordinated action → precision approaches individualize management → population health and policy mechanisms support scale, sustainability and equity.

Conceptual coherence was assessed by determining whether each domain had a distinct primary function and logically defensible relationships with the others. Conceptual completeness was considered in relation to the major stages of translation:

Risk identification → coordinated action → individualized management → implementation and population impact.

Domains that substantially duplicated another component were treated as cross-cutting enablers rather than independent domains.

### 2.7 Expert involvement and limitations of the methodology

No Delphi process, nominal group technique or formal international expert consensus exercise was undertaken. The framework was developed through author-led literature synthesis, conceptual mapping, comparison with existing models and iterative refinement. Accordingly, PM2.0 is presented as a literature-informed, theoretically derived and hypothesis generating conceptual framework, not as a validated consensus model.

## 3 Conceptual evolution and the distinctive proposition of PM2.0

The term “Periodontal Medicine 1.0” is not an established term in the literature. In this article it is used retrospectively as a heuristic description of the earlier association oriented development of periodontal medicine. The designation “Periodontal Medicine 2.0” similarly represents an author derived metaphor for a proposed translational direction rather than a formally recognized historical transition.

The earlier periodontal medicine paradigm was scientifically essential. It expanded attention from local periodontal pathology toward systemic associations and stimulated research into biological mechanisms (1, 3). Subsequent evidence synthesis, classification systems and clinical guidelines strengthened the clinical foundations of periodontal care (37–39).

Several existing frameworks already address components of the proposed model. Integrated oral-healthcare approaches address the separation of dentistry from primary healthcare (27–31). Interprofessional education and collaborative practice frameworks address communication and professional coordination (40–44). Precision medicine provides a basis for individualized prevention and treatment (45, 46). Digital health and AI frameworks address information exchange, decision support and remote care (47–53). Population health and universal health coverage approaches position oral health within broader health policy (32–36) (Table 1).

**Table 1:** Distinction between PM2.0 and related concepts.

Existing concept	Principal contribution	Limitation relative to PM2.0	Added dimension of PM2.0
Traditional periodontal medicine	Oral-systemic associations and biological mechanisms	Primarily association and evidence oriented	Connects sufficiently mature evidence with implementation
Periodontal-systemic medicine	Relationships between periodontal and systemic disease	Does not necessarily specify a delivery architecture	Provides a structured evidence to care pathway
Integrated oral healthcare	Integration of dental and medical services	Primarily service integration	Links integration with screening, personalization, technology and scale
Interprofessional collaborative care	Communication and coordinated management	Primarily focuses on professional coordination	Places collaboration within a wider periodontal pathway
Precision dentistry/oral health	Individualized assessment and treatment	Primarily patient level	Connects personalization with screening, collaboration and implementation
Person-centred periodontal care	Preferences, participation and shared decisions	Primarily patient-centred	Positions patient participation across the entire system
Digital health/AI	Prediction, communication and monitoring	May be evaluated primarily as a technological capability rather than as part of an integrated care pathway	Treats technology as enabling infrastructure
Population health frameworks	Prevention, equity and system implementation	Generally not periodontal specific	Connects periodontal care with implementation, sustainability and equitable scale-up
PM2.0	Integrates these functions around the periodontal patient	Not yet empirically validated	Explicit functional relationships, feedback, evidence calibration and testability

Thus, the domains represent distinct but interconnected functions rather than sequential or independent components. Their overlap is intentional and reflects functional interdependence, while each domain retains a distinct primary function.

The distinctive proposition of PM2.0 therefore lies in the functional relationships between the domains rather than in their individual content. The relationships are bidirectional, with feedback from clinical and population level information. The domains are not interchangeable and are not expected to contribute equally at every stage of care. Weakness at one point in this architecture can limit the value of the others. For example, screening without referral produces information without action, precision without demonstrated clinical utility increases data burden without necessarily improving care, and digital adoption without implementation safeguards may increase inequity without improving outcomes.

The periodontal patient remains the clinical anchor, while evidence based periodontal treatment remains the therapeutic foundation. Systemic and health system components are incorporated only when they are sufficiently supported, clinically relevant, actionable and appropriate to the patient's care.

## 4 The PM2.0 framework

### 4.1 Integrated oral-systemic screening

The primary function of integrated screening is identification of actionable risk and appropriate escalation.

PM2.0 does not propose universal systemic screening for every periodontal patient. It proposes risk based and context specific screening when a clinically meaningful condition, an appropriately evaluated screening method, a confirmatory pathway and an appropriate professional role are available.

Diabetes provides a particularly relevant example because of the established bidirectional relationship between periodontitis and diabetes and the availability of validated risk assessment and chairside screening approaches (10, 11). Cardiometabolic risk assessment may similarly include blood pressure, smoking status, obesity related measures and other established risk factors when appropriately incorporated into local care pathways.

By contrast, associations involving neurodegenerative disease and pregnancy should not automatically generate routine systemic screening. Evidence concerning the oral-brain axis remains predominantly associative and mechanistic, with causality and preventive benefit from periodontal intervention unresolved (16–21). Similarly, the existence of an association between periodontitis and adverse pregnancy outcomes should not be interpreted as proof that periodontal treatment prevents adverse obstetric outcomes (22–25).

Screening must also be distinguished from diagnosis. A positive screening result is an actionable risk signal, not a diagnosis. Dental professionals should perform only those screening activities supported by training, professional scope, regulation and local healthcare pathways.

The operational sequence is therefore:

Eligible patient → validated risk assessment/screening → positive result → documented referral → confirmatory diagnosis → appropriate management → feedback to dental care where relevant.

The principal implementation measures are screening reach, positive findings, referral initiation, referral completion and diagnostic follow through.

### 4.2 Interprofessional collaborative care

Screening creates value only when appropriate action follows. Interprofessional care therefore provides the mechanism connecting periodontal findings with broader healthcare.

Collaboration should not mean transferring responsibility for systemic disease to dental professionals. Rather, responsibilities should be explicitly defined. Dental professionals may identify risk, provide periodontal care and communicate relevant findings; medical professionals remain responsible for diagnosis and management of systemic conditions within their scope.

The diabetes example illustrates reciprocal collaboration: dental professionals can identify periodontal disease and potentially recognize individuals requiring metabolic assessment, while medical teams can recognize the importance of periodontal evaluation in patients with diabetes (10, 11).

Effective collaboration requires defined referral pathways, secure information exchange, feedback mechanisms and shared decision making. Interprofessional education is therefore an important enabling strategy (40–44).

The key distinction is between referral and collaboration. Referral is an event; collaboration is an ongoing mechanism for coordinated care. The principal output of this domain is therefore coordinated care, not merely referral.

### 4.3 Precision periodontal medicine

Precision periodontal medicine is defined here as the use of validated patient specific biological, clinical, behavioural, environmental and social information to improve periodontal prevention, treatment or maintenance decisions. It is therefore narrower than precision dentistry, which encompasses individualized approaches across dental specialties.

Precision does not imply that more data automatically produce better care. Biomarkers, microbiome profiles, genomic information and predictive models should enter clinical practice only when they demonstrate incremental value over established periodontal assessment.

The oral microbiome provides an important research opportunity because periodontitis reflects complex interactions between microbial communities and host responses (54–57). Salivary and other biomarkers may similarly provide additional information regarding periodontal or systemic processes (58, 59). However, association with disease does not establish that a marker improves clinical decision making.

Behavioural and social determinants are integral to this definition. Tobacco use, health literacy, socioeconomic circumstances, treatment preferences and access to care may substantially influence periodontal outcomes and should not be displaced by molecular data.

### 4.4 Population health and policy integration

Individual clinical innovations cannot produce population benefit without supportive systems. Periodontitis is highly prevalent, and its distribution is influenced by socioeconomic conditions, behaviours and access to care (30, 31, 60). These determinants overlap with those of major non-communicable diseases, supporting integration through common risk factor and population health approaches.

WHO strategies increasingly recognize oral health as an integral component of universal health coverage and health system planning (32, 33). Policy integration may include workforce development, preventive financing, oral health surveillance, interprofessional education and interoperable information systems (34–36).

However, population integration should follow evidence rather than precede it. A promising intervention should demonstrate effectiveness, feasibility, safety, cost-effectiveness and equity before large scale adoption.

### 4.5 Cross-cutting enablers

Digital health and AI, patient engagement, health literacy, equity, ethics, and implementation science were positioned as cross-cutting enablers because they support and influence all four core domains.

Digital technologies provide infrastructure for linking patients, clinicians and health systems. Electronic health records can support information exchange, while telehealth can facilitate access and specialist communication (47–49). AI may assist with image interpretation, classification and risk prediction, but technical performance should not be equated with clinical utility (50–52).

Digital technologies may enhance education, reminders and monitoring, but digital solutions can also widen disparities where access or literacy is limited. Equity should therefore be evaluated alongside effectiveness.

A model demonstrating high accuracy in a controlled dataset does not necessarily demonstrate external validity, appropriate clinical use, cost-effectiveness or improved outcomes. Digital technologies also introduce risks, including privacy concerns, cybersecurity, algorithmic bias, unequal access and automation related errors (61, 62).

Periodontal care requires sustained patient engagement, self-management, behavioural change and supportive periodontal maintenance. Health literacy influences understanding of disease risk, treatment recommendations and preventive behaviours, while patient participation and self-care are important components of sustained periodontal management (63, 64).

Evaluation of a PM2.0 pathway should assess not only periodontal parameters but also patient reported quality of life, treatment satisfaction, health literacy, adherence, access and experience of coordinated care.

Implementation science provides a structured approach to assessing contextual determinants and implementation processes, helping determine whether PM2.0 can be integrated effectively into routine care. Ethical and governance considerations should also be incorporated throughout implementation and evaluation (Figure 1).

**Figure 1:**
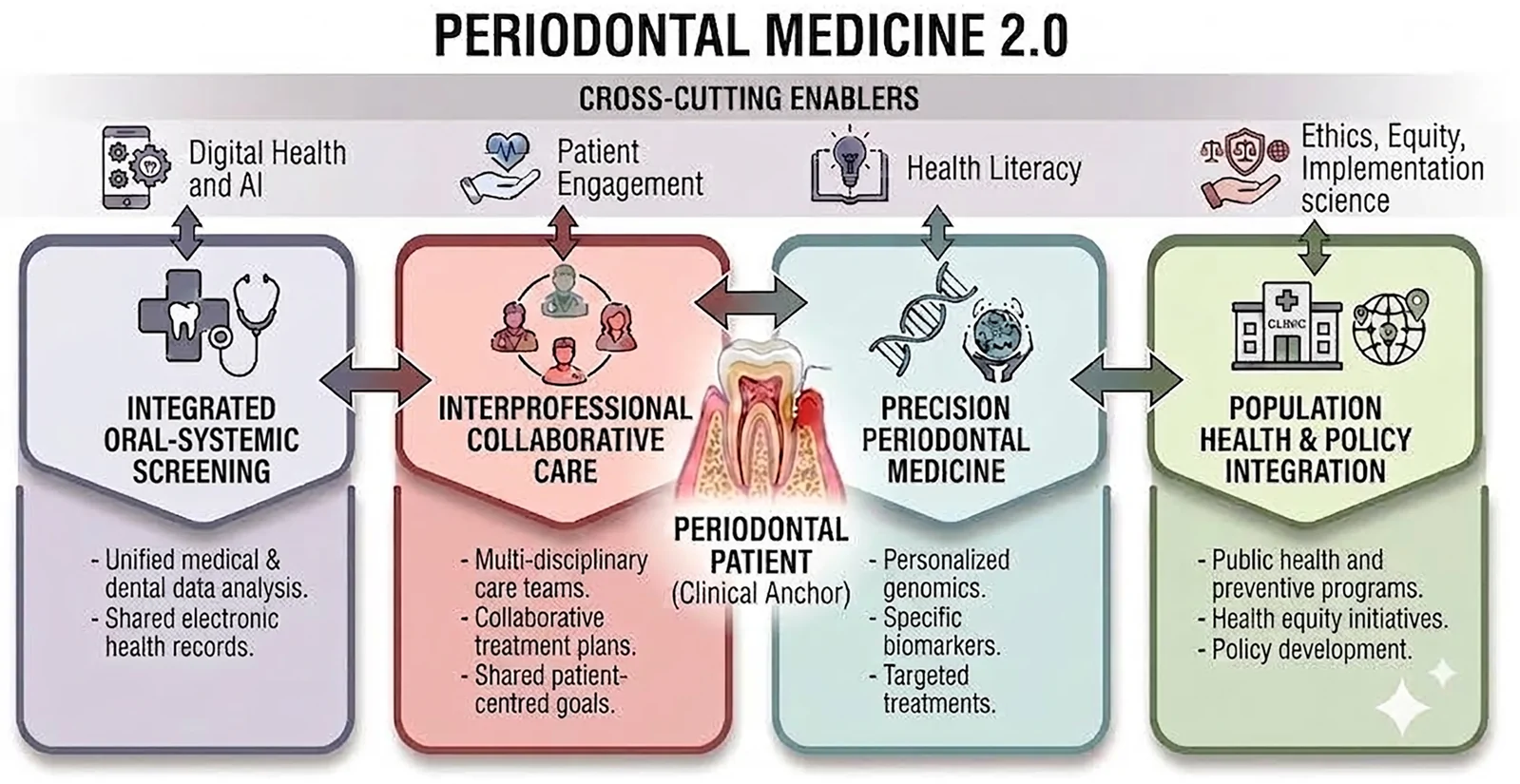
PM2.0: a framework for evidence-calibrated, integrated and patient-centred care.

## 5 An operational pathway for PM2.0

The framework is intended to be tested through a patient-centred longitudinal pathway, not adopted as a universal screening protocol. The framework proposes a risk based sequence.

### 5.1 Stage 1: periodontal entry point

Comprehensive periodontal assessment establishes periodontal diagnosis, stage, grade, complexity, risk factors and treatment needs according to contemporary periodontal standards (37, 38).

### 5.2 Stage 2: selective systemic risk assessment

Only patients meeting predefined risk or eligibility criteria undergo relevant screening. The selected tool depends on the condition, evidence, training, regulation and local resources.

### 5.3 Stage 3: actionable result

A positive screening result identifies potential risk and should prompt appropriate follow-up assessment rather than be interpreted as a diagnosis.

### 5.4 Stage 4: referral and coordination

The patient is referred through a defined pathway. Referral completion and diagnostic outcomes are recorded where feasible.

### 5.5 Stage 5: individualized periodontal care

Established periodontal treatment remains the therapeutic foundation (26). Additional patient specific information is incorporated only when it demonstrates clinical value.

### 5.6 Stage 6: patient participation

Health literacy, shared decision making, behavioural support, self-care and supportive periodontal care are integrated throughout the pathway.

Emerging work conceptualizing periodontal pockets as chronic wounds requiring continuous management reinforces the importance of sustained clinician patient participation and repeated management rather than viewing periodontal treatment as a single event (63). This remains an evolving perspective rather than a new established treatment standard.

### 5.7 Stage 7: digital monitoring

Digital tools may support communication, education, monitoring and decision support where appropriate.

### 5.8 Stage 8: reassessment and adaptation

Periodontal, patient reported and relevant systemic outcomes are reassessed, and care is modified according to evidence and patient circumstances.

Assess → risk stratify → screen selectively → refer → coordinate → individualize → empower → monitor → reassess.

## 6 Evidence maturity, implementation and safeguards

A central principle of PM2.0 is that the four domains differ in their current evidence maturity, clinical readiness and implementation status. The framework therefore does not treat all domains as equally established. Some components are supported by established clinical or policy practice, whereas others remain emerging or research oriented (Table 2).

**Table 2:** Evidence maturity of PM2.0 domains.

PM2.0 domain	Current evidence status
Integrated oral-systemic screening	Established for selected conditions; emerging for broader applications
Interprofessional collaborative care	Established healthcare principle; periodontal specific outcome evidence emerging
Precision periodontal medicine	Emerging; predominantly research stage for biomarker/omics guided care
Population health and policy integration	Established policy direction; periodontal specific implementation evidence emerging

### 6.1 Overall interpretation

Taken together, the evidence supports a mixed-maturity model rather than a uniformly established four domain framework. Selected oral-systemic risk identification is relatively mature; interprofessional collaboration and population health integration have strong conceptual and policy foundations but less periodontal specific outcome evidence; and precision periodontal medicine remains substantially dependent on further validation and clinical utility research.

A useful evidence calibration pathway is:

Biological plausibility → clinical evidence → validation → clinical utility → implementation → patient benefit → population impact.

Accordingly, the level of integration should remain proportional to evidence maturity. Established interventions may inform routine care; emerging approaches should undergo appropriate validation and, where sufficiently mature, implementation evaluation; and research stage or conceptual applications should remain within appropriately governed research.

This distinction is particularly important for neurodegeneration, pregnancy outcomes, biomarkers, microbiome profiling, genomics and AI, where biological plausibility, association or technical performance does not by itself establish causality or intervention benefit.

### 6.2 Implementation and safeguards

Implementation should be evaluated using established implementation science approaches, including assessment of contextual determinants and implementation outcomes. Potential unintended consequences should be evaluated alongside potential benefits. These include overmedicalization, false-positive findings, unnecessary referrals, workforce burden, financial costs, algorithmic bias, privacy risks and inequitable access. Integration should therefore increase coordination without increasing unnecessary intervention.

PM2.0 proposes five safeguards before broader implementation:

1. Is the evidence sufficient for the proposed action?
2. Is the activity within professional and regulatory scope?
3. Is there a defined pathway for acting on the information?
4. Do anticipated benefits outweigh harms and costs?
5. Can effectiveness, safety and equity be measured?

If these conditions are not met, the application should remain within appropriately governed research or pilot evaluation.

## 7 Measuring success

The framework becomes testable only when outcomes are defined.

Periodontal outcomes: probing depth, clinical attachment level, bleeding on probing, periodontal inflamed surface area, periodontal stability, tooth loss and treatment response.

Patient outcomes: oral health related quality of life, satisfaction, health literacy, shared decision making, adherence and supportive care attendance.

Systemic/healthcare outcomes: appropriate screening, detection of previously unrecognized risk, referral completion, diagnostic follow through and relevant systemic outcomes when specifically evaluated.

Implementation outcomes: acceptability, adoption, appropriateness, feasibility, fidelity, reach, implementation cost and sustainability (65).

Equity outcomes: differences in access, uptake and outcomes across socioeconomic, geographic and digital access groups.

Economic outcomes: healthcare utilization, resource use and cost-effectiveness.

The framework should not be considered successful merely because screening rates or technology adoption increase. Success requires meaningful benefit without unacceptable harm, cost or inequity. Effectiveness and cost-effectiveness should therefore be evaluated through prospective implementation studies comparing the model with usual care, using predefined clinical, patient-centred, health system, economic and implementation outcomes.

## 8 Testable propositions and research priorities

This theoretical proposition concerns whether integration itself adds value.

### 8.1 Proposition 1: integrated screening

Integrated oral-systemic screening will provide greater clinical value when linked to defined referral, follow-up and feedback pathways than when screening is implemented without coordinated care.

### 8.2 Proposition 2: precision care

The clinical value of precision periodontal approaches will depend on whether additional biological, behavioural or digital information produces incremental improvement in clinical decision making and patient outcomes beyond conventional assessment.

### 8.3 Proposition 3: system level implementation

The population level impact of periodontal medicine will depend on the ability to translate effective clinical pathways into sustainable, scalable and equitable health system models.

### 8.4 Proposition 4: central hypothesis

Integration of the four functionally differentiated domains of PM2.0 will provide greater translational value than isolated implementation of individual components, after accounting for implementation context, resource requirements and potential harms.

This central proposition can be tested by comparing integrated and fragmented models of care across clinical, patient reported, implementation, economic, safety and equity outcomes. The propositions therefore position PM2.0 as a testable theoretical framework rather than a descriptive model.

## 9 Discussion

PM2.0 responds to a specific translational problem: periodontal medicine has accumulated substantial scientific knowledge, yet the mechanisms through which that knowledge can be converted into coordinated, measurable healthcare remain incompletely developed. The framework does not suggest that association research has become obsolete. Associations, biological mechanisms and causal investigations remain essential to evaluating evidence maturity. Rather, PM2.0 introduces an additional translational question: when is evidence sufficiently mature, clinically relevant and actionable to justify integration into care?

This distinction is particularly important where evidence remains uncertain, such as the oral-brain axis. Associations between periodontitis and cognitive or neurodegenerative outcomes, together with plausible inflammatory and microbial mechanisms, justify continued investigation but do not establish causality or demonstrate that periodontal treatment prevents neurodegeneration (16–21). Similarly, maintaining oral health during pregnancy is clinically important, but whether periodontal intervention improves obstetric outcomes remains a separate empirical question (22–25). These examples illustrate why PM2.0 applies evidence calibration rather than uniform integration across oral-systemic conditions.

The same principle applies to precision periodontal medicine. Biomarkers, genomic information and AI do not constitute precision care simply because they provide additional patient level data. Precision should therefore be judged by clinical utility rather than data volume.

Digital integration should likewise not be regarded as inherently beneficial. Interoperability, AI and telehealth are enabling technologies whose value depends on demonstrated clinical utility, implementation feasibility, safety, privacy and equity. This reinforces the rationale for treating digital health and AI as cross-cutting enablers rather than independent core domains.

PM2.0 also differs from a general integrated oral health model because the periodontal patient remains its clinical anchor. The framework incorporates systemic healthcare only where it is relevant, actionable and appropriately supported by evidence. It therefore does not seek to redefine dentistry as medicine or expand the professional responsibilities of dental practitioners beyond their scope. This bounded approach is important for limiting overmedicalization, unnecessary screening and inappropriate referrals.

The framework generates a focused research agenda. Future studies should determine whether selective screening in dental settings improves appropriate detection and referral; whether structured dental medical collaboration improves patient outcomes; whether precision approaches provide incremental value beyond conventional assessment; whether AI improves clinical decisions and patient outcomes rather than classification accuracy alone; and whether integrated models remain feasible, affordable, safe and equitable in routine care.

Implementation science is consequently an integral evaluation mechanism rather than a separate future direction. Implementation studies can examine contextual determinants, implementation outcomes, sustainability, equity and economic consequences. The Consolidated Framework for Implementation Research (CFIR) provides one established approach for examining contextual factors that may influence implementation (65, 66).

The framework has several limitations. It is conceptual rather than empirically validated and was not developed through formal Delphi or international expert consensus. Its four domains are therefore provisional, and their relative importance may vary across healthcare systems. Some proposed applications, particularly those involving precision technologies and broader oral-systemic screening, remain research stage. PM2.0 also cannot prescribe universal screening tools or referral pathways because professional scope, regulation, workforce capacity and available resources differ across settings.

These limitations are consistent with the intended purpose of the article. PM2.0 is not presented as a clinical guideline or established model of care, but as a literature-informed, testable conceptual hypothesis.

## 10. Conclusion

Periodontal Medicine 2.0 is an author derived conceptual hypothesis proposing a potential evolution of periodontal medicine from predominantly association oriented research toward evidence-calibrated translation and integrated, patient-centred care.

PM2.0 hypothesizes that appropriately connected and evidence-calibrated functions may generate greater translational value than fragmented approaches. This proposition remains unvalidated and requires empirical testing across clinical, patient reported, implementation, economic and equity outcomes.

The proposed transition is therefore not simply from association to integration, but from association to evidence based action and from action to demonstrable impact.
